# Pomegranate juice intake enhances the effects of aerobic training on insulin resistance and liver enzymes in type 2 diabetic men: a single-blind controlled trial

**DOI:** 10.1186/s40795-022-00538-3

**Published:** 2022-05-17

**Authors:** Sasan Nemati, Vahid Tadibi, Rastegar Hoseini

**Affiliations:** Department of Exercise Physiology, Faculty of Sport Sciences, Razi University, Kermanshah, Iran

**Keywords:** Aerobic training, Pomegranate, Diabetes mellitus, Liver diseases, Glycemic indexes, Integrative Medicine, Complementary and alternative Medicine

## Abstract

**Background:**

Lifestyle interventions are the first-line treatment for Type 2 diabetes mellitus (T2DM), highly prevalent in the community. This study aimed to examine the 8-week separate and combined effects of aerobic training (AT) and pomegranate juice intake (PJI) on insulin resistance and serum levels of liver enzymes, liver enzymes, and insulin resistance in men with T2DM.

**Methods:**

This study evaluated the alterations of anthropometric indices, insulin resistance, and liver enzymes in 40 middle-aged men (40–50) with T2DM. Participants were randomly assigned into four groups: AT+PJI (*n* = 10); AT (*n* = 10); PJI (*n* = 10), and control (C) (*n* = 10). The AT program consisted of 60–75% of maximum heart rate (HR_max_), 40–60 min/day, and 3 days/wk. Participants in the PJI group consumed 240 ml of pomegranate juice (sugar or additive-free) daily for 8 weeks.

**Results:**

AT+PJI, PJI, and AT groups decreased anthropometric indices, HOMA-IR, and liver enzymes after 8 weeks. In contrast, the C group significantly increased the mentioned variables after the intervention. The result showed that AT+PJI significantly lowered liver enzymes, anthropometric indices, and HOMA-IR than AT or PJI alone. Also, the results of this study showed no significant difference between AT and PJI groups. However, in these groups, significant improvements in these variables were observed compared to the control group.

**Conclusion:**

Due to the effect of combined AT+PJI in improving T2DM risk factors, it could be recommended for T2DM patients to prevent increased liver enzymes and insulin resistance.

## Background

Type 2 Diabetes Mellitus (T2DM) is the most common health problem [1], with more than 415 million cases worldwide [2]. T2DM might increase the levels of alanine aminotransferase (ALT), aspartate aminotransferase (AST), and gamma-glutamyl transferase (GGT). This is mainly caused by increased oxidative stress in tissues and can also be caused by increased blood sugar levels [3, 4]. It has been reported that pomegranate juice has antioxidant properties and can be used for therapeutic purposes [5]. Pomegranate juice may enhance antioxidant defenses and reduce the oxidative damage induced by diabetes [6]. In addition to counteracting the adverse effects of oxidative stress caused by T2DM, pomegranate juice can reduce the index of cell membrane damage and increase serum antioxidant capacity and nitric oxide, which is the essential anti-inflammatory and antioxidant agent in vascular endothelium [5, 7]. Also, Human and cellular studies have shown that pomegranate juice can increase the expression and activity of the Paraoxonase 1 enzyme [5, 7], which has antioxidant properties and protects LDL -C and HDL-C against oxidation [5, 7].

It is known that diet and medication alone are not sufficient in treating and controlling blood glucose and lipid metabolism in human models of diabetes, and physical activity should be added to the daily schedule of these individuals [8]. It has also been shown that a sedentary lifestyle is significantly correlated with the mortality rate in T2DM patients [8, 9]. Regular exercise reduces T2DM risk factors and improves blood sugar regulation [10, 11], fat oxidation, and obesity in humans [12]. Studies in both healthy volunteers and T2DM patients engaged in a supervised long-term exercise program show enhanced antioxidant capacity and prevented cell destruction by protecting cells against the harmful effects of oxidative stress [13, 14]. Different types of exercise, including aerobic, resistance, and stretching training, can be prescribed for diabetic patients [15]; However, aerobic training (AT) is considered essential for managing T2DM [16]. There are various reports regarding the effects of physical activity on liver enzymes in human studies [17, 18].

Some studies have investigated the different effects of AT and pomegranate juice intake (PJI) on T2DM [19–21]. However, the concurrent effects of these two interventions on the liver enzymes of type 2 diabetic patients are not fully understood. Therefore, we hypothesized that the PJI would have an additional favorable effect when combined with exercise on the serum concentration of liver enzymes and insulin resistance. This research contributes to our previous findings, which focused primarily on the general health and quality of life in T2DM patients.

## Methods

### Experimental approach

This study was a single-blinded, placebo-controlled, randomized clinical trial among 40 T2DM patients aged 40–50 years that was registered on the website of the Iranian clinical trial at http://www.irct.ir: IRCT20200907048650N1. This research was conducted according to the Declaration of Helsinki. The Research Ethics Committees approved the study protocol of Kermanshah Razi University (IR.RAZI.REC.1398.007); informed consent was taken from all patients.

### Subjects

**Fig. 1:**
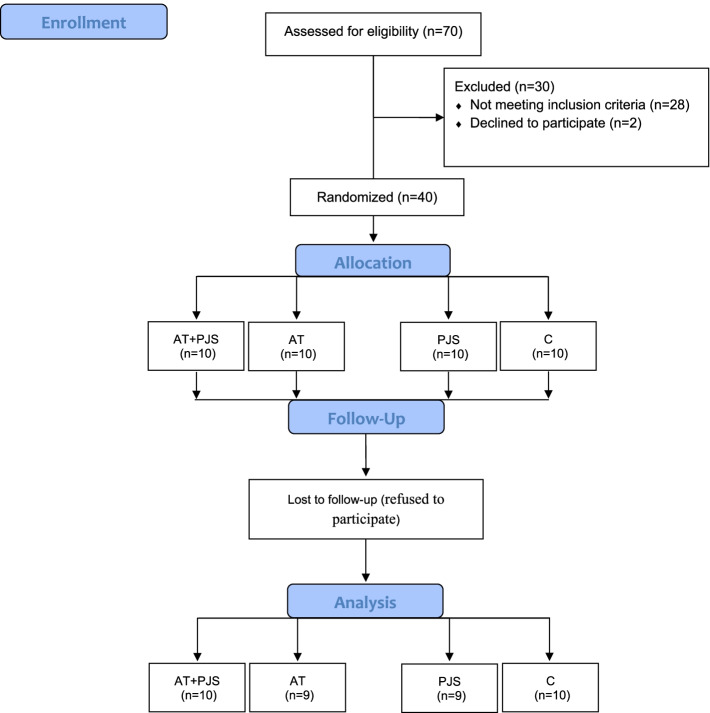
Flow chart of the study population

The sample size was evaluated to be 9 in each group using G. POWER 3.1 software (alpha error = 0.05, statistical power = 0.80, and effect size = 0.70). Due to the probability of refusal to continue the study, forty men (10 in each group) with T2DM, aged 40–50 years, were randomly selected. The voluntary sampling method was used among patients from the Diabetes Medical & Health Center, Kermanshah, Iran. Then subjects were randomly (using a random-numbers table) divided into four groups; AT+PJI (*n* = 10), AT (*n* = 10), PJI (*n* = 10), and control (C) group (*n* = 10). One participant in the AT group and one subject in the PJI group refused to continue the intervention program. (Fig. 1). All patients were allowed to leave the study at any point.

Inclusion criteria included: men with T2DM and no other chronic disease or musculoskeletal problems, aged 40–50 years, fasting blood sugar above 126 mg, taking just one table of Metformin 500 mg per day, no insulin injection, body mass index above 24.99, no smoking, and lack of regular exercise for at least last 6 months before the beginning of the study.

### Intervention

#### Aerobic training

The 8-week exercise training program consisted of 40–60 minutes of jogging and running at 60–75% of maximum heart rate (HRmax) (Table 1). All of the training protocols were supervised by the exercise physiologists and were executed on a track. Exercise training consists of three phases: warm-up (10 min), AT (20–40 min), and cool down (10 min). The AT phase began with 20 minutes of jogging at 60% of HR_max_ in the first week. Then, it progressed to 40 minutes of running at 75% of HR_max_ in the final week. Each participant underwent heart-rate monitoring with a polar heart rate monitor (model: FT1) and used the 6–20 rating of perceived exertion (RPE) scale (Table 1) to assure that the desired heart rate (exercise intensity) was achieved and maintained during the AT phase [12]. The HRmax formula determined the target heart rate [HR_max_ = 220− age]. The AT program was based on previous studies and the American Diabetes Association (ADA) [22, 23].

**Table 1:** Aerobic training protocol

Variables	Week							
	1	2	3	4	5	6	7	8
**Intensity (**HR_Max_**)**	60–65%	60–65%	65–70%	65–70%	65–70%	70–75%	70–75%	70–75%
**Time (min)**	40	40	50	50	50	60	60	60
**Borg scale**	10	10	11	11	11	12	12	13

#### Supplementation

Participants in AT+PJI and PJI consumed 240 ml of pure pomegranate juice from Orum Narin Company’s SHADLEE brand (without sugar and other additives) daily after lunch for 8 weeks. The Orum Narin Company confirmed that all juice bottles contain the same amount of energy and nutrients. A detailed description of the preparation of the pomegranate juice is reported elsewhere [24]. Participants in the AT and C groups received a water-based non-pomegranate placebo with the same shape (juice container), color, and smell (made by Orum Narin Company’s SHADLEE brand as requested).

### Measurements

Before the intervention, participants were thoroughly acquainted with the aims of the study, were taught to fill out the requested questionnaires appropriately, and received the necessary oral and written explanation. First, demographic data, health status, current alcohol intake, and medication were recorded from all participants. Before attending the training program, the participants filled out a detailed semi-quantitative food frequency questionnaire tailored to the Iranian population. It included everyday food items, portion sizes, and meals to record and analyze 3-day food recalls before and after the intervention. The Food Processor nutritionist four software (FPN4) was used to determine the food intake and macronutrient consumption (protein, fat, and carbohydrates). The participants were asked to consume the same food and macronutrient composition 1 day before collecting blood samples in the pre and post-tests. The participants’ diet consisted of 55% carbohydrates, 30% fat, and 15% protein.

#### Anthropometric and body composition

Three days before the intervention, height was measured to the nearest 0.5 cm using a stadiometer (DETECTO, Model 3PHTROD-WM, USA). Bodyweight (BW), Body mass index (BMI), waist-hip ratio (WHR), and body fat percentage (BFP) were obtained by a bioelectric impedance body composition analyzer (Zeus 9.9 PLUS: Jawon Medical Co., Ltd., Kungsang Bukdo, South Korea) in the fasting state.

#### Blood sampling

Participants were asked to refrain from strenuous exercise 1 week before the blood sampling. In the laboratory, a blood sample of 10 ccs was taken from a brachial vein, centrifuged at 3000 rpm for 10 minutes, followed by serum isolation. The serums from blood samples were frozen at − 28 °C for testing. After a 12-hour fast, blood samples were taken in two phases, 48 hours before and after the first and last training sessions. Serum concentrations of liver enzymes (ALT, AST, GGT) were measured using the Enzymatic Method with Autoanalyzer Kit (Kenza 120, Biolabo Diagnostics, France). Fasting blood sugar was calculated using the enzymatic method using Pars Azmoon test kits (Iran), insulin by ELISA method using the Mercoda Company kit (Sweden), and Insulin resistance index was measured using the following formula [25].

$$\mathrm{HOMA}-\mathrm{IR}=\kern0.75em \left[\mathrm{Fasting}\ \mathrm{insulin}\ \left(\upmu \mathrm{U}/\mathrm{ml}\right)\times \mathrm{Fasting}\ \mathrm{glucose}\ \left(\mathrm{mmol}/\mathrm{l}\right)\right]/405$$

### Statistical analysis

The results were analyzed using IBM SPSS (version 24.0). The descriptive statistics quantity was presented as the mean and the standard error of the mean (SE). The Shapiro–Wilk’s test evaluated the normality of distribution. One-way ANOVA and t-test were used to compare the mean hepatic risk factors between and within groups. Tukey’s post hoc test was used if significant differences were found.

## Results

This study was performed from November 2020 to February 2021. The findings on some demographic information and anthropometric indices of the participants and their between-group comparison are presented in Table 2. Based on the results of the t-test, there were significant differences in the mean of BW, BMI, BFP, and WHR after the intervention compared to the pre-test. After 8 weeks, BW, BMI, BFP, and WHR significantly decreased in AT+PJI (*P* = 0.001, *P* = 0.001, *P* = 0.001, and *P* = 0.001), AT (*P* = 0.001, *P* = 0.018, *P* = 0.001, and *P* = 0.001), and PJI (*P* = 0.003, *P* = 0.033, *P* = 0.016, and *P* = 0.001), while in the C, these variables increased significantly (*P* = 0.001, *P* = 0.028, *P* = 0.001, and *P* = 0,002) (Table 2).

**Table 2:** Mean ± SD of the Variables before and after the intervention among the groups

Variables	AT + PJI (*n* = 9)	AT (*n* = 10)	PJI (*n* = 9)	C (*n* = 10)	*P*-Value ^a^
Age (years)	43.22 ± 2.48	42.60 ± 1.89	41.33 ± 1.50	41.81 ± 1.88	0.336
Height	162 ± 54.18	166 ± 5.45	164.55 ± 4.79	164.45 ± 5.78	0.393
Body Weight (Kg)					
Before	90.44 ± 4.92	88.80 ± 3.35	88.44 ± 2.50	90.09 ± 4.20	
After	85.22 ± 2.81	85.20 ± 3.45	86.22 ± 2.77	92.45 ± 5	
P†	0.001*	0.001*	0.003*	0.001*	
Δ	−5.22 ± 2.11 ^**β**^	−3.60 ± 0.10 ^**β**^	− 2.22 ± 0.02 ^**β**^	2.36 ± 0.80	0.001 ¥
BMI (kg/m^2^)					
Before	34.55 ± 3.14	32.28 ± 1.78	32.69 ± 1.30	33.34 ± 1.51	
After	32.54 ± 2.25	30.97 ± 1.73	31.86 ± 1.13	34.22 ± 1.88	
P†	0.001*	0.018*	0.033*	0.028*	
Δ	−2.01 ± 0.89 ^**β**^	−1.31 ± 0.05 ^**β**^	−0.86 ± 0.17 ^**β**^	0.88 ± 0.37	0.002 ^**¥**^
Body Fat Percent (%)					
Before	37.55 ± 3.04	36.10 ± 3.41	35.33 ± 3.31	35.27 ± 3.52	
After	31.66 ± 3.53	31.10 ± 3.84	34.11 ± 2.61	36.81 ± 3.76	
P†	0.001*	0.001*	0.016*	0.001*	
Δ	−5.89 ± 0.49 ^**β**^	−5 ± 0.43 ^**β**^	−1.22 ± 0.70 ^**β**^	1.54 ± 0.24	0.002 ^**¥**^
WHR					
Before	0.99 ± 0.06	0.98 ± 0.04	0.97 ± 0.03	0.98 ± 0.02	
After	0.95 ± 0.02	0.96 ± 0.01	0.95 ± 0.02	0.99 ± 0.03	
P†	0.001*	0.001*	0.001*	0.002*	
Δ	−0.04 ± 0.04 ^**β**^	−0.02 ± 0.03 ^**β**^	−0.02 ± 0.01 ^**β**^	0.01 ± 0.01	0.028 ^**¥**^

*AT + PJI* Aerobic training + pomegranate juice intake group, *AT* Aerobic training group, *PJI* pomegranate juice intake group, *C* the control group

*Data analysis was done by the analysis of one-way analysis of variance (ANOVA) and least significant difference post-hoc Tukey’s test after adjustment for baseline values; P†: Statistical analysis was done by paired sample t-test

*: Significantly different in comparison pre and post within the groups

¥: Significantly different comparing Δ between groups

β: Significantly different compared with C

The results of one-way ANOVA showed significant differences in the above variables between the groups in the post-test. The results of Tukey’s post hoc test show no significant difference in the BW, BMI, BFP, and WHR between AT+PJI, AT, and PJI. However, significant differences were found in the BW and BMI in AT+ PJI, AT, and PJI compared to the C (Table 2).

No between-group or within-group significant differences were observed in the carbohydrate, lipid, and protein intake during the 8-week intervention period (Table 3).

**Table 3:** Mean ± SD of the nutrients intakes before and after the intervention

CHO intake (gm/d)					
Before	523.11 ± 23.16	530.44 ± 43.17	515.24 ± 23.27	528.16 ± 16.29	
After	520.32 ± 17.21	528.22 ± 34.36	514.45 ± 13.12	532.23 ± 37.19	
P†	0.231*	0.264*	0.689*	0.201	
Δ	−2.79 ± 5.95	−2.22 ± 8.81	−0.79 ± 10.15	4.07 ± 20.90	0.054
Proteins intake (gm/d)					
Before	125.16 ± 5.06	127.21 ± 3.03	124.11 ± 5.03	125.15 ± 6.32	
After	124.12 ± 3.23	126.26 ± 4.13	123.24 ± 4.16	126.12 ± 4.06	
P†	0.223*	0.293*	0.384*	0.321	
Δ	1.04 ± 1.83	0.95 ± 1.10	−0.87 ± 0.87	0.97 ± 2.26	0.086
Lipids intake (gm/d)					
Before	96.14 ± 4.16	98.33 ± 2.28	94.13 ± 6.25	93.57 ± 3.11	
After	95.05 ± 3.08	97.36 ± 3.21	93.24 ± 2.34	94.16 ± 4.08	
P†	0.216*	0.253*	0.361*	0.454	
Δ	−1.09 ± 1.08	−0.97 ± 0.93	−0.89 ± 3.91	0.59 ± 0.97	0.068
Energy intake (Kcal/d)					
Before	3458.34 ± 50.10	3515.57 ± 68.44	3404.57 ± 56.48	3455.37 ± 39.47	
After	3433.21 ± 36.49	3494.16 ± 60.95	3389.92 ± 30.06	3480.84 ± 67.24	
P†	0.117	0.183	0.215	0.108	
Δ	−25.13 ± 13.61	−21.41 ± 7.49	−14.65 ± 26.42	25.47 ± 27.77	0.052

*AT + PJI* Aerobic training + pomegranate juice intake group, *AT* Aerobic training group, *PJI* pomegranate juice intake group, *C* the control group

*Data analysis was done by the analysis of one-way analysis of variance (ANOVA) and least significant difference post-hoc Tukey’s test after adjustment for baseline values; P†: Statistical analysis was done by paired sample t-test

*: Significantly different in comparison pre and post within the groups

¥: Significantly different comparing Δ between groups

β: Significantly different compared with C

There were significant differences in FBS, insulin, and HOMA-IR in post-test compared to pre-test in AT+PJI (*P* = 0.001, *P* = 0.001, and *P* = 0.001), AT (*P* = 0.001, *P* = 0.021, and *P* = 0.024), and PJI (*P* = 0.001, *P* = 0.042, and *P* = 0.043) (Table 4). The results of one-way ANOVA showed significant differences in the FBS, insulin, and HOMA-IR. Significant differences were observed in FBS, insulin, and HOMA-IR in AT+PJI, AT, and PJI compared to the C (Table 4). Also, there were significant differences in FBS (*P* = 0.001, *P* = 0.001), insulin (*P* = 0.012, *P* = 0.001), and HOMA-IR (*P* = 0.007, *P* = 0.001) in the AT+PJI group compared to the AT and PJI groups alone; respectively. Furthermore, the results revealed significant differences between AT and PJI groups in FBS (*P* = 0.001), insulin (*P* = 0.024), and HOMA-IR (*p* = 0.022).

**Table 4:** Comparison of Mean ± SD of FBS, insulin, and HOMA-IR within and between the groups

Variables	AT + PJI (*n* = 9)	AT (*n* = 10)	PJI (*n* = 9)	C (n = 10)	*P*-Value ^a^
FBS (mg/dl)					
Before	144.44 ± 3.12	145.10 ± 3.84	146.88 ± 7.42	146.90 ± 7.24	
After	126.44 ± 3.50	133.10 ± 3.95	139.33 ± 6.34	149.09 ± 7.53	
P†	*P* = 0.001*	*P* = 0.001*	*P* = 0.001*	*P* = 0.002*	
Δ	−18 ± 0.38 ^**μ€β**^	−12 ± 0.11 ^**€β**^	−7.55 ± 1.08 ^**β**^	2.19 ± 0.29	0.001 ^**¥**^
Insulin (μU/ml)					
Before	7.30 ± 0.70	7.31 ± 0.86	7.06 ± 0.75	7.30 ± 0.78	
After	6.65 ± 1.09	7 ± 0.87	6.95 ± 0.76	7.38 ± 0.90	
P†	*P* = 0.001*	*P* = 0.021*	*P* = 0.042*	*P* = 0.049*	
Δ	−0.65 ± 0.39 ^**μ€β**^	−0.31 ± 0.01 ^**€β**^	−0.11 ± 0.01 ^**β**^	0.08 ± 0.12	0.039 ^**¥**^
HOMA-IR					
Before	2.60 ± 0.27	2.62 ± 0.30	2.56 ± 0.29	2.65 ± 0.37	
After	2.08 ± 0.33	2.30 ± 0.25	2.39 ± 0.26	2.72 ± 0.40	
P†	*P* = 0.001*	*P* = 0.024*	*P* = 0.043*	*P* = 0.049*	
Δ	−0.52 ± 0.06 ^**μ€β**^	−0.32 ± 0.05 ^**€β**^	−0.17 ± 0.03 ^**β**^	0.07 ± 0.03	0.026 ^**¥**^
AST (U/L)					
Before	37.88 ± 0.74	38.23 ± 0.68	36.22 ± 1.20	36.48 ± 1.20	
After	26.33 ± 0.70	32.33 ± 0.16	33.85 ± 0.78	38.01 ± 1.09	
P†	*P* = 0.001*	*P* = 0.024*	*P* = 0.038*	*P* = 0.045*	
Δ	−11.55 ± 0.04 ^μ€β^	−5.90 ± 0.52^€β^	−2.37 ± 0.42^β^	1.53 ± 0.11	0.001 ^**¥**^
ALT (U/L)					
Before	39.07 ± 4.01	38.80 ± 3.21	36.77 ± 3.27	36.20 ± 3.02	
After	29.11 ± 2.42	33.85 ± 2.17	32.55 ± 3.21	39.76 ± 3.20	
P†	*P* = 0.001*	*P* = 0.012*	*P* = 0.025*	*P* = 0.039*	
Δ	−9.96 ± 1.59^μ€β^	−4.95 ± 1.04 ^€β^	−4.22 ± 0.06^β^	3.56 ± 0.18	0.001 ^**¥**^
GGT (U/L)					
Before	38.22 ± 2.53	39.22 ± 2.68	38.33 ± 3.39	39.18 ± 2.63	
After	29.44 ± 1.66	34.01 ± 2.36	34.22 ± 2.58	41.45 ± 3.35	
P†	*P* = 0.001*	*P* = 0.010*	*P* = 0.027*	*P* = 0.041*	
Δ	−8.78 ± 0.87^μ€β^	−5.21 ± 0.3^€β^	−4.11 ± 0.81 ^β^	2.27 ± 0.72	0.011 ^**¥**^

*AT + PJI* Aerobic training + pomegranate juice intake group, *AT* Aerobic training group, *PJI* pomegranate juice intake group, *C* the control group

*Data analysis was done by the analysis of one-way analysis of variance (ANOVA) and least significant difference posthoc Tukey’s test after adjustment for baseline values; P†: Statistical analysis was done by paired sample t-test

*: Significantly different in comparison pre and post within the groups

¥: Significantly different comparing Δ between groups

μ: Significantly different compared with AT

€: Significantly different compared with PJI

β: Significantly different compared with C

There were significant differences in the AST, ALT, and GGT between the pre-test and post-test in AT+PJI (*P* = 0.001, *P* = 0.001, and *P* = 0.001), AT (*P* = 0.024, *P* = 0.012, and *P* = 0.010), and PJI (*P* = 0.038, *P* = 0.025, and *P* = 0.027), as detailed in Table 4, The results of one-way ANOVA showed no significant differences in AST, ALT, and GGT between the groups in the pre-test. However, there were significant differences in liver enzymes between the groups in the post-test; AST, ALT, and GGT decreased significantly in AT+PJI, AT, and PJI compared to C. Also, the result shows significantly lower ALT and GGT (*P* = 0.029, and *P* = 0.001, respectively) in AT+PJI compared to AT and significantly lower GGT (*P* = 0.001) in AT+PJI compared to PJI.

## Discussion

The effects of 8 weeks of separate and combined PJI and AT on insulin resistance and liver enzymes were investigated in this study. We hypothesized that the PJI would have additional favorable effects on insulin resistance and liver enzymes when combined with exercise. Our hypothesis was supported by the findings, as evidenced by a significant decrease in insulin resistance, liver enzymes, and anthropometric indices. The results showed that 8 weeks of AT+PJI significantly improved BW, BMI, BPF, and WHR in men with T2DM. However, PJI and AT also significantly affected anthropometric indices, but these improvements were better in AT+PJI. Based on the previous findings, increased BFP is highly correlated with fat accumulation in the liver and T2DM [26, 27]. AT improved anthropometric indices probably by boosting daily energy intake and fat oxidation in skeletal muscles, resulting in fat loss, particularly in the abdominal area [28, 29].

Anthropometric indices of participants with T2DM decreased after PJI; Despite numerous scientific studies showing various therapeutic properties of pomegranate [30, 31], few scientific studies focused on the anti-obesity properties in T2DM patients. In line with the present study’s findings, Lei et al. (2007) reported that obese rats fed a high-fat diet showed considerable weight loss, energy intake, and adipose tissue following treatment with pomegranate extract [29]. Also, Shukla et al. (2008) reported that consumption of pomegranate extract significantly reduced the appetite of obese mice fed a high-fat diet [32]. The anti-obesity property of pomegranate juice might be attributable to its anti-lipase and lipolytic activity. Also, the continents, including punicalagin, hydrolyzable tannins, anthocyanins, and ellagic acid, serve anti-obesity properties [7, 33].

Furthermore, after 8 weeks of AT+PJI, HOMA-IR decreased significantly; a similar decrease was found after AT and PJI alone. However, the combined effect of AT+PJI was significantly more potent than separate. Exercise increases insulin sensitivity in T2DM patients by activating metabolic pathways related to non-insulin-related glucose transport and inducing structural adaptations (such as muscle regeneration and angiogenesis) [22, 34]. The stimulation of the peroxisome proliferator-activated receptor-coactivator (PGC)-1 pathway is another possible mechanism of the effect of AT, A pathway that is diminished in T2DM [23, 35]. Also, it has been shown that the peroxisome proliferator-activated receptor gamma (PPAR-γ) pathway is affected by the punicic acid continent of pomegranate juice which in turn, modulates glucose uptake and diabetes-induced inflammation [36]. Based on Olvera-Sandoval et al. (2022) study, the antidiabetic content found in pomegranates helps reduce blood sugar levels in T2DM patients [37].

This study showed that 8 weeks of combined AT+PJI significantly decreased the liver enzymes (ALT, AST, GGT) in the AT+PJI, AT, and PJI compared to the control group. This decrease was significantly higher in AT+PJI than in AT or PJI alone. Pomegranate juice might decrease the activity of ALT, AST, and GGT enzymes probably by reducing blood glucose, glycation of antioxidant enzymes, and increasing the clearance of ROS [37]. As pomegranate juice contains many polyphenolic and flavonoid compounds, it has strong antioxidant properties and prevents oxidative stress in the body [38, 39]. Therefore, it seems that enhancing the antioxidant system of diabetic patients following PJI might help improve and prevent diabetes complications [40, 41].

Small sample size, not evaluating HbA1c index, single blind design, and not checking the nutritional profile of the pomegranate juice were among the limitations of the present study.

## Conclusions

Finally, both separate or combined AT and PJI improve anthropometric indices, insulin resistance, and liver enzymes in men with T2DM. However, combined AT+PJI induces more favorable improvements. Due to the effect of combined AT+PJI in improving T2DM risk factors, it could be recommended to T2DM patients to prevent increased liver enzymes and insulin resistance in these.

## Data Availability

All data generated during this study are included in this published article.

## Abbreviations

AT: Aerobic Training
PJI: Pomegranate Juice Intake
HR_max_: Maximum Heart Rate
ALT: Alanine Aminotransferase
AST: Aspartate Aminotransferase
GGT: Gamma-Glutamyl Transferase
BW: Bodyweight
BMI: Body mass index
WHR: Waist-Hip Ratio
BFP: Body Fat Percentage
